# Repair of Lafosse I subscapularis injury adds no additional value in anterosuperior rotator cuff injury

**DOI:** 10.1186/s12891-021-04805-5

**Published:** 2021-11-03

**Authors:** Binghao Zhao, Qingsong Zhang, Bo Liu

**Affiliations:** Department of Osteoarthrosis, Renmin Hospital, Hubei University of Medicine, No.39 Middle Chaoyang Raod, Maojian District, Shiyan, 442000 Hubei China

**Keywords:** Lafosse I subscapularis injury, Anterosuperior rotator cuff injury

## Abstract

**Background:**

The study aimed to explore the additional value of repair of Lafosse I subscapularis injury compared with debridement in anterosuperior rotator cuff injury.

**Methods:**

The prospective study was conducted on a total of 41 patients with supraspinatus tendon tear combined with Lafosse I subscapularis injury. Eighteen patients were divided into the repair group and 23 patients were divided into the non-repair group. The two groups were compared for intraoperative parameters, pain score, range of motion of the shoulder joint, shoulder joint function and quality of life (QoL) at pre-operation, 3 and 6 months postoperatively and the final follow-up visit.

**Results:**

The width of supraspinatus tendon tear did not exceed 3 cm and did not retract beyond the glenoid in among patients. There was no statistical difference of preoperative data between two groups, including age, course of disease, positive Jobe test, positive Bear-hug test, positive Lift-off test, Patte stage, longitudinal tear and pain severity (*P* > 0.05). Compared to preoperative levels, the severity of pain, ASES scores and EQ-5D-3L scores were significantly lower at 3 and 6 months postoperatively and the final position (*P* < 0.05). However, there was no statistical difference in pain severity, ASES scores and EQ-5D-3L scores between repair group and non-repair group (*P* > 0.05). Similarly, compared to preoperative levels, the range of motion of shoulder joint was significantly improved after operation, including internal rotation, external rotation, forward flexion and elevation (*P* < 0.05). However, there was no statistical difference in range of motion of shoulder joint between repair group and non-repair group (*P* > 0.05).

**Conclusion:**

Operative treatment can effectively lessen severity of pain in the patients, improve shoulder joint function, increase the range of motion of the shoulder joint and enhance the QoL in treating anterosuperior rotator cuff injury. However, repair of subscapularis brings no benefit compared to debridement in treating supraspinatus tendon tear combined with Lafosse I subscapularis injury.

## Introduction

Over the recent years, the incidence of rotator cuff injury is approximately 30% in persons aged above 60 years and rotator cuff injury has been an important social issue with the acceleration of population aging [1]. Rotator cuff injury mainly manifests clinically as weakness, limited range of motion and pain of the shoulder joint, severely impacting on the daily life of patients and reducing their quality of life (QoL) [2]. Previous studies consider that the incidence of subscapularis injury is low and open surgery was main method in clinical treatment, but it is difficult to diagnose Lafosse I and II subscapularis injury [3]. With the arthroscopic techniques being used widely in clinic, the diagnostic rate of partial subscapularis injury is increasing. Evidence indicates that up to 50% patients with rotator cuff injury have concurrent subscapularis injury [4]. In 2007, Lafosse categorized subscapularis injury into type I, II, III, IV and V, which is still in widely use clinically [5]. Surgical treatment of Lafosse II-V can achieve a satisfactory outcome [6]. Previous studies showed that if Lafosse I injury is not promptly and effectively treated, the shoulder joint pain and the rotator cuff tear will be likely aggravated [7]. However, some studies also show that Lafosse I injuries require merely intraoperative debridement without reconstruction [8]. Therefore, treatment for Lafosse I subscapularis injury remains controversial and lacks consensus. Therefore, this study analyzed patients with supraspinous tendon tear combined with Lafosse I subscapularis injury, in order to explore the addittional value of repair of Lafosse I subscapularis injury in anterosuperior rotator cuff injury.

## Materials and methods

### Clinical data

This prospective study involved 46 patients with supraspinatus tendon tear combined with Lafosse I subscapularis injury who received treatment at our hospital between January 2018 and November 2019. Since 5 patients were lost to follow-up, 41 patients with supraspinatus tendon injury combined with Lafosse I subscapularis injury were enrolled in this study at last. Based on concealed random allocation from a computer-generated random numbers table, 18 patients were assigned to the repair group and 23 patients to the non-repair group. In the repair group, the age ranged from 43 to 76 years, with a mean age of (58.2 ± 9.1) years. There were 7 males and 11 females. The course of the disease ranged from 1 to 7 months, with a mean course of (3.2 ± 0.5) months. In the non-repair group, the age ranged from 44 to 77 years, with a mean age of (58.9 ± 9.2) years. There were 10 males and 13 females. The course of the disease ranged from 1 to 7 months, with a mean course of (3.1 ± 0.5) months. The two groups were comparable in the baseline variables (*P* > 0.05). The study protocol followed the Declaration of Helsinki of the World Medical Association.

Inclusion criteria: Clinical diagnosis of supraspinatus tendon tear combined with type I subscapularis injury utilizing pre-operative MRI; partial tear is not more than superior 1/3 of subscapularis tendon; shoulder pain, weakness, and limited range of motion; written informed consent; no cervical disease or brachial plexus injury; complete clinical data; good compliance; mentally normal; consent to arthroscopic repair of rotator cuff injury and intraoperative repair of the subscapularis. Exclusion criteria: a history of shoulder dislocation and surgery; radiological evidence of rheumatoid arthritis or osteoarthritis of the shoulder joint; bilateral shoulder joint disease, and patients uncooperative with postoperative rehabilitation; diseases of the cardiac, cerebral and pulmonary systems, and patients who cannot tolerate surgery; coagulation abnormalities; pregnant or lactating women.

### Methods

#### Preoperative preparation

All patients received cervical plexus block plus brachial plexus block followed by general anesthesia. The patient was placed in the lateral decubitus position on the healthy side, and the axillary region was cushioned. If preoperative diagnosis included frozen shoulder, it was first gently manipulated under anesthesia. Intraoperative systolic blood pressure was maintained at 95–110 mmHg (1 mmHg = 0.133 kPa).

#### Surgical methods

The standard 30**°** arthroscope was inserted via the traditional posterior portal to examine the glenohumeral joint, and a shaver and a plasma knife were entered via the anterior portal to debride the soft tissues around the coracoid process. Injury of the subscapularis tendon and tendon of the long head was evaluated. Supraspinatus tendon tear was graded using Patte classification, and subscapularis injury was categorized using Lafosse method. Tendon tear, semi-dislocation, and dislocation of the long head of the biceps brachii were recorded. The articular side of the coracoid process was exposed and coracoplasty was performed if the distance between the coracoid process and the humerus was < 4 mm.

#### The non-repair group

**Fig. 1:**
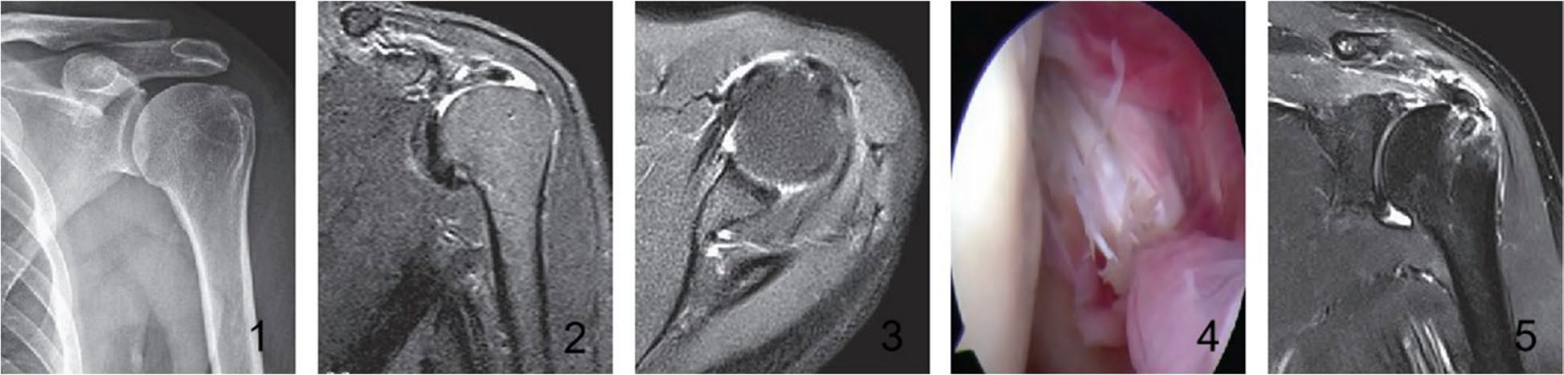
Note: 1–1: Preoperative anteroposterior radiograph shows an elevated acromion; 1–2: Preoperative oblique MRI T2WI of the left shoulder joint reveals a supraspinous tendon tear, Patte stage 2 retraction; 1–3: Preoperative transverse MRI T2WI of the left shoulder joint shows mixed hyperintensities; 1–4: Injury of the upper 1/3 of the subscapularis tendon (Lafoss I) was seen under the arthroscope intraoperatively, non-repaired; 1–5: MRI T2WI of the left shoulder joint at the final follow-up visit reveals an anchor in the greater tuberosity of the humerus and continuous recovery of the supraspinous tendon

The supraspinatus tendon tear was debrided using a shaver or a plasma knife. After debridement of hyperplasic synovium and scar tissues, the subscapularis tendon required no repair. The tendon of the long head required no treatment in SLAP type I and II injury and with no longitudinal tear, abrasion, and dislocation in the long head of the biceps brachii tendon. In SLAP III and above injuries and longitudinal tear, abrasion, instability, and dislocation of the long head of the biceps brachii tendon, the tendon of the long head was transected, and the intertubercular groove was stabilized with anchors. In persons aged above 70 years and nonphysical workers, the tendon of the long head was transected without stabilization. The supraspinatus tendon was repaired by using suture anchors in a single or double row technique (Fig. 1).

#### The repair group

**Fig. 2:**
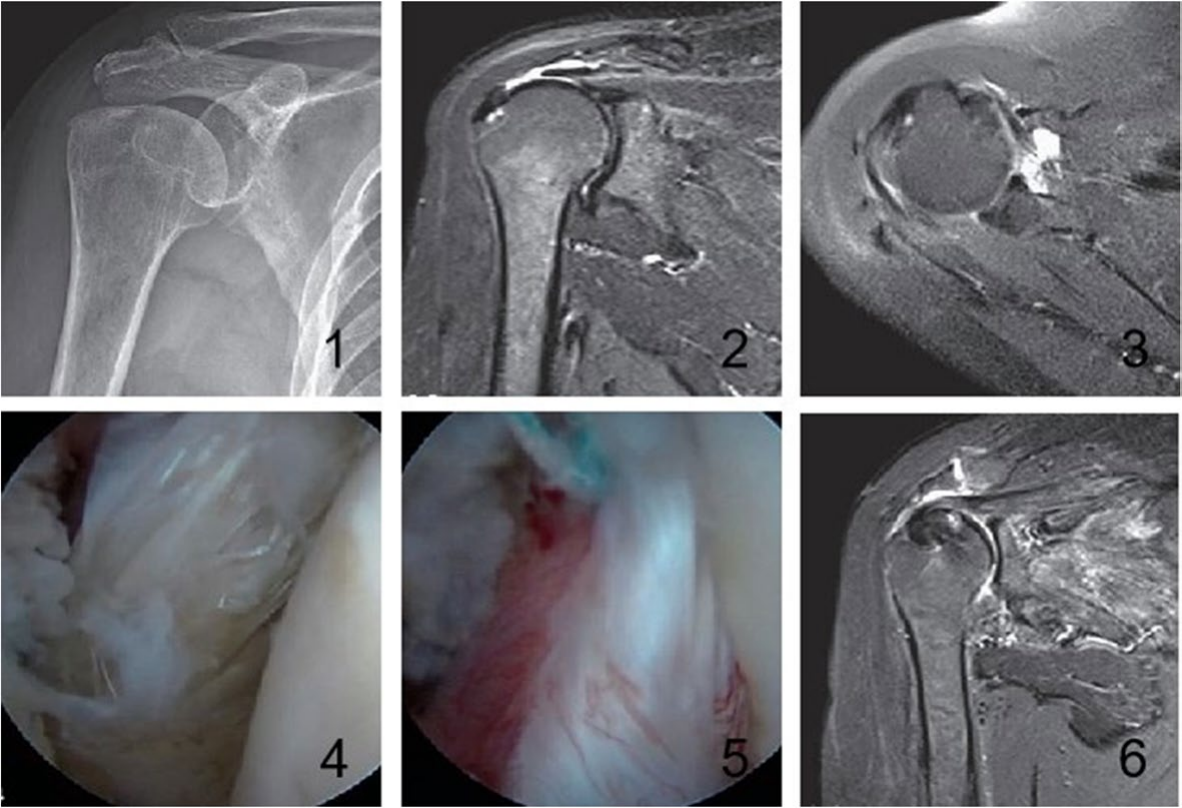
Note: 2–1: preoperative anteroposterior radiograph shows increased bone density of the acromion and greater tuberosity; 2–2: Preoperative oblique MRI T2WI of the shoulder joint shows supraspinous tendon tear, Patte stage 2 retraction; 2–3: Preoperative transverse MRI T2WI of the shoulder joint reveals mixed hyperintensities in the subscapularis tendon; 2–4: Intraoperative arthroscopy shows injury in the upper 1/3 of the subscapularis tendon (Lafosse I); 2–5: Intraoperative arthroscopy demonstrates subscapularis tendon Lafosse I injury. Repair of the subscapularis tendon was undertaken; 2–6: MRI T2WI in the oblique view at the final follow-up visit reveals an anchor in the greater tuberosity of the humerus and continuous recovery of the supraspinatus tendon

For repair of the subscapularis, a shaver and plasma knife were used to debride the abrasion in the medial subscapularis tendon and a small amount of cartilage in the medial subscapularis tendon footprint in the lesser tuberosity was removed and milled with a burr. A 5.0 mm suture anchor (Smith & Nephew, USA) was placed and sutured using a suture hook and secured with knots. The tendon of the long head required no treatment in SLAP type I and II injury and with no longitudinal tear, abrasion, semi-dislocation and dislocation in the long head of the biceps brachii tendon. In SLAP III and above injuries and longitudinal tear, abrasion, instability, and dislocation of the long head of the biceps brachii tendon, the tendon of the long head was transected, and the intertubercular groove was stabilized with anchors. In persons aged above 70 years and nonphysical workers, the tendon of the long head was transected without stabilization. The supraspinatus tendon was repaired with anchors (Fig. 2).

#### Postoperative recovery

Postoperatively, the shoulder joint in all patients was stabilized with suspension with an abduction orthosis for 4–6 weeks. The shoulder was only engaged in passive motion within 6 weeks postoperatively and thereafter active motion and muscle training at 12 weeks postoperatively.

### Observational parameters

Intraoperative condition of the two groups was observed, and severity of pain, range of motion and function of the shoulder joint and QoL of life were compared at pre-operation, 3 and 6 months postoperatively and the final follow-up visit.

#### Severity of pain

Pain was evaluated using the visual analog scale (VAS) at at pre-operation, 3 and 6 months postoperatively and the final follow-up visit [9]. The scale has a score 0 to 10 and higher scores indicate more severe pain.

#### The range of motion of the shoulder joint

The range of internal and lateral external rotation and active shoulder joint forward flexion and elevation were recorded. For examination of the range of internal rotation, the patients were told to put the hand of the affected side behind the back with the palm facing back and the thumb moving up the spinous process as much as possible and the most proximal spinous process reached by the thumb was the range of internal rotation. For examination of the range of external rotation, both arms naturally drooped at the sides of the body, the elbow joint flexed to 90°, the palm faced up, and the patient was told to externally rotate the arm using the upper arm as the axel. The maximal angle between the arm and the sagittal plane was recorded. For examination of active shoulder joint forward flexion and elevation, the patient was in the upright position, the elbow joint was extended, and the palm faced medially. The upper arm being examined was actively elevated in the sagittal plane, and the angle between the torso and the arm maximally elevated was recorded.

#### Shoulder joint function

Shoulder joint function before and after treatment was evaluated using American Shoulder and Elbow Surgeons Shoulder Score (ASES) [10]. The scale includes a physician-rated component and a patient-reported component, but the final score is only calculated using the patient-reported component. The subjective rating by the patient includes activities of daily living (50%) and pain (50%), with a total score of 100. Higher scores indicate better shoulder joint function.

#### QoL

QoL was evaluated using three-level EuroQol five-dimensions (EQ-5D-3L) before and after treatment [11]. It includes 5 dimensions: anxiety or depression, pain or discomfort, daily activities, self-care, and mobility. Each dimension has a core 1–3, and the total score ranges from 0 to 100 and higher scores indicate better overall health.

### Statistical analysis

Data was analyzed using SPSS 21.0 software (IBM Corp, Chicago, IL, USA) and all date was expressed in means ± SD. Independent t tests were performed to assess differences in change of age, operative time and ASES score. The Mann-Whitney U tests were performed to assess differences in change of severity of pain, shoulder joint function and EQ-5D-3L scores. *P* < 0.05 represented significant difference.

## Results

### Comparison of general and intraoperative data in the two groups

There was no statistical difference in age, gender, course of illness, positive Jobe test, positive Bear-hug test and positive Lift-off test between the two groups (*P* > 0.05) (Table 1). Among them, the width of supraspinatus tendon tear did not exceed 3 cm and did not retract beyond the surface of the glenohumeral joint. There was no statistical difference in Patte stage, longitudinal tear of the long head of the biceps brachii tendon, semi-dislocation, abrasion, and dislocation of the tendon of the long head, and operative time (*P* > 0.05) (Table 2).

**Table 1:** Comparison of general data in the two groups

Variables	Repair group (*n* = 18)	Non-repair group (*n* = 23)	*P*-value
Age (years)	58.23 ± 9.09	58.91 ± 9.17	0.814
Sex (male/female)	7/11	10/23	0.767
Course of disease (months)	3.13 ± 0.53	3.09 ± 0.51	0.808
Positive Jobe test (n)	8	10	0.732
Bear-hug test (n)	5	6	0.753
Positive Lift-off test (n)	6	9	0.885
Positive Neer sign (n)	11	12	0.350
Positive Hawkins test (n)	8	12	0.853
History of trauma (n)	10	13	0.767
Preoperative X rays (n)	14	20	0.934

**Table 2:** Comparison of intraoperative data in the two groups

Variables	Repair group (*n* = 18)	Non-repair group (*n* = 23)	*P*-values
Patte stage (n)			
I	8	10	0.951
II	10	13	0.951
Longitudinal tear of the biceps brachii tendon (n)	4	4	0.698
Semi-dislocation of the tendon of the long head (n)	2	2	0.698
Abrasion (n)	4	4	0.796
Dislocation (n)	1	1	0.859
Operative time (min)	156.93 ± 33.83	137.93 ± 31.29	0.070

### Comparison of severity of pain in the two groups

There was no statistical difference in the severity of pain preoperatively in the two groups (*P* > 0.05). Compared to the severity of pain at pre-operation, the severity of pain was significantly lower after operation in both groups (*P* < 0.05). However, there was no statistical difference in the severity of pain between repair and non-repair group after operation, including 3 and 6 months postoperatively and the final position (*P* > 0.05) (Table 3).

**Table 3:** Comparison of severity of pain in the two groups ($\overline{x}\pm \mathrm{s}$)

Time	Repair group (*n* = 18)	Non-repair group (*n* = 23)	*P*-values
Preoperative	3.23 ± 0.87	3.19 ± 0.82	0.881
3 months postoperatively	2.52 ± 0.56	2.61 ± 0.67	0.649
6 months postoperatively	1.32 ± 0.32	1.35 ± 0.31	0.763
Final follow-up visit	0.91 ± 0.23	0.96 ± 0.21	0.472
*P*-values	<0.001^*^	<0.001^*^	

*P < 0.05 versus preoperatively

### Comparison of shoulder joint function and the range of motion in the two groups

There was no statistical difference in preoperative ASES score between the two groups (*P* > 0.05). The ASES scores after operation were lower than the preoperative ASES score in both groups, and the difference was statistically significant (*P* < 0.05). There was no statistical difference in range of internal and external rotation and the active range of forward flexion and elevation of the shoulder joint between repair group and the non-repair group before surgery (*P* > 0.05). The range of motion of the shoulder joint at post-operation were lower than those before surgery in both groups, and the difference was statistically significant (*P* < 0.05). However, there was no statistical difference between two groups in the ASES score and the range of motion of the shoulder joint after operation, including 3 and 6 months postoperatively and the final position (*P* > 0.05) (Tables 4 and 5).

**Table 4:** Comparison of shoulder joint function ($\overline{x}\pm \mathrm{s}$)

Time	Repair group (*n* = 18)	Non-repair group (*n* = 23)	*P*-values
Preoperative	42.38 ± 6.21	42.98 ± 6.29	0.762
3 months postoperatively	62.12 ± 7.53	63.11 ± 8.17	0.693
6 months postoperatively	76.93 ± 6.78	75.76 ± 6.71	0.584
Final follow-up visit	90.29 ± 7.01	88.97 ± 7.21	0.559
*P*-values	<0.001^*^	<0.001^*^	

*P < 0.05 versus preoperatively

**Table 5:** Comparison of the range of motion of the shoulder joint in the two groups ($\overline{x}\pm \mathrm{s}$)

Variables	Time	Repair group (*n* = 18)	Non-repair group (*n* = 23)	*P*-values
Range of internal rotation	Preoperative	L5 (lateral thigh to L4)	L5 (lateral thigh to L4)	–
	3 months postoperatively	L3 (L3-L5)	L3(L2-L1)	–
	6 months postoperatively	L2 (L5-L1)	L3 (L5-L1)	–
	Final follow-up visit	L1 (L3-T11)	L2 (L4-T11)	–
Lateral range of external rotation (°)	Preoperative	29.73 ± 3.92	29.92 ± 3.98	0.879
	3 months postoperatively	31.41 ± 4.13	32.04 ± 4.02	0.625
	6 months postoperatively	36.33 ± 4.29	34.98 ± 4.18	0.316
	Final follow-up visit	44.23 ± 4.76	42.28 ± 4.81	0.203
	*p*-values	<0.001*	<0.001*	
Active shoulder joint forward flexion and elevation (°)	Preoperative	96.93 ± 12.98	97.34 ± 13.02	0.788
	3 months postoperatively	124.3 ± 11.24	126.01 ± 10.21	0.616
	6 months postoperatively	142.92 ± 14.39	145.93 ± 15.02	0.520
	Final follow-up visit	149.83 ± 16.83	151.29 ± 17.01	0.785
	*p*-values	<0.001*	<0.001*	

*P < 0.05 versus preoperatively

### Comparison of QoL between the two groups

There was no statistical difference in preoperative EQ-5D-3L scores between the two groups (*P* > 0.05). The EQ-5D-3L scores at post-operation were lower than the preoperative EQ-5D-3L score in both groups, and the difference was statistically significant (*P* < 0.05). There was no statistical difference in EQ-5D-3L scores between the two groups at at post-operation, including 3 and 6 months postoperatively and the final position (*P* > 0.05) (Table 6).

**Table 6:** Comparison of QoL between the two groups ($\overline{x}\pm \mathrm{s}$)

Time	Repair group (*n* = 18)	Non-repair group (*n* = 23)	*P*-value
Preoperative	63.29 ± 6.82	64.93 ± 6.93	0.456
3 months postoperatively	70.76 ± 5.98	74.08 ± 5.23	0.021
6 months postoperatively	76.93 ± 7.32	78.82 ± 7.28	0.024
Final follow-up visit	79.07 ± 7.29	80.38 ± 7.32	0.037
*P*-values	<0.001*	<0.001*	

*P < 0.05 versus preoperatively

## Discussion

Rotator cuff injury is a common shoulder joint disease. Increasing patients with rotator cuff injury receive appropriate diagnosis and treatment, with the aging of the population and increased knowledge of the condition by both the physician and patients. Previous study has pointed out that compared to simple subscapularis tear patients, subscapularis tear combined with supraspinatus tendon tear is more common [12], which was defined as anteroinferior rotator cuff injury [13]. In this study, we found that operative treatment can effectively lessen severity of pain in the patients, improve shoulder joint function, increase the range of motion of the shoulder joint and enhance the QoL in treating anterosuperior rotator cuff injury. However, repair of subscapularis brings no benefit compared to debridement in treating supraspinatus tendon tear combined with Lafosse I subscapularis injury.

Prolonged and repetitive traction and friction of subscapularis tendon cause tendon edema, nonbacterial inflammation and rupture, leading to persistent pain the anterior shoulder, limiting the range of motion and causing functional abnormalities, thus severely reducing the life quality of patients [14]. Currently, arthroscopic repair has become one of the major surgical treatment modalities for the disease, which has the advantages of less invasiveness, fast postoperative recovery and good long-term outcome [15]. A study by Wamer et al. [13] in 19 cases of anterosuperior rotator cuff tear showed that after 40 months of postoperative follow up, the subjective results were excellent in 5 cases, good in 3 cases, satisfactory in 4 cases, and poor in 7 cases, and pointed out that the postoperative outcome of subscapularis injury combined with supraspinatus tendon or infraspinatus injury was relatively poor. The study by Gerber et al. [11] showed that after repair of the subscapularis, the external rotation of the shoulder joint was limited, especially following open surgery. However, with the promotion of arthroscopic techniques, the arthroscope has been widely used in various types of surgery clinically. Arthroscopic treatment of subscapularis injury in combination with effective postoperative rehabilitation training can alleviate the limited range of motion of the shoulder joint. The study by Jiang et al. [16] revealed that arthroscopic subscapularis loosening for simple subscapularis injury could markedly relieve shoulder pain, increase the range of motion of the shoulder joint and improve shoulder joint function; meanwhile, it has no effect on the power of shoulder joint muscles. These results suggests that operative treatment with arthroscopic techniques can effectively improve the shoulder joint function in subscapularis injury, especially Lafosse II-V subscapularis injury.

Katthagen et al. [17] treated 65 cases of anterosuperior rotator cuff injury by local debridement of Lafosse I subscapularis injury after repair of the supraspinatus tendon, and the patients were followed up for 12 months postoperatively, and the results showed that the treatment effectively increased shoulder joint function of the patients, reduced pain scores and lessened the severity of pain. A most recent study by Neviaser et al. [18] of 57 cases of anterosuperior rotator cuff tear, including 39 patients who received arthroscopic repair, 18 cases who underwent open surgical repair showed that with a mean follow up duration of 34 months, the range of motion of the shoulder joint and ASES scores increased markedly postoperatively. The current study showed the ASES scores and range of motion of the shoulder joint were lower in both groups at post-operation than their preoperative counterparts. Meanwhile, the pain severity at post-operation were lower in both groups than the preoperative counterparts. These results suggests that operative treatment can effectively improve the shoulder joint function and lessen the the pain severity in Lafosse I subscapularis injury, including debridement and repair. However, there is still much controversy clinically on whether Lafosse I subscapularis injury should be repaired or not, especially in those with concurrent supraspinatus tendon tear, and it remains inconclusive whether surgical treatment has any effect on the overall surgical outcome [19].

Previous study has pointed out that subscapularis tear combined with supraspinatus tendon tear will change the kinematic mechanics of shoulder joint [20]. Since repair of subscapularis tear can not restore kinematic mechanics of shoulder joint, arthroscopic repair of subscapularis tear is not promoted in Lafosse I subscapularis injury. However, Gausden et al. [21] believed that the upper part of supraspinatus tendon bears more tension and surgical repair should be performed in Lafosse I subscapularis injury. If subscapularis tear is not repaired, the prognosis is not definite in Lafosse I subscapularis injury. Some studies speculate that the integrity of rotator cuff is easy to be damaged without repair of subscapularis tear and surgical repair can restored the tension and medial structure of shoulder joint. In addition, the subscapularis tendon is involved in the suspension bridge of the shoulder joint [22]. During repairing supraspinatus tendon injury, the subscapularis injury should been repair in anterosuperior rotator cuff tears, which is beneficial to lessen the imbalance of shoulder’s suspension bridge and protect the supraspinatus tendon in turn. However, these conclusion still lack enough and definite clinical and radiographic evidence.

A retrospective analysis by Bartl et al. [6] of 48 cases of anterosuperior rotator cuff tear who were followed up for 48 months revealed that the postoperative shoulder joint function markedly improved, but at the final follow up, not all patients had fully recovered rotator cuff muscle power. Wirth et al. [8] treated 73 cases of anterosuperior rotator cuff injury, 41 cases received surgical treatment and 32 cases underwent subscapularis repair and the results showed that the operative time in the non-repair subscapularis group was shorter, and there was no marked difference in shoulder joint function at 6 and 24 months postoperatively between the two groups. Consistent with previous, the current study showed that there is no significant difference between repair and non-repair groups in the pain severity, ASES scores and range of motion of the shoulder joint, suggesting that repair of Lafosse I subscapularis injury brings no benefit in treating anterosuperior rotator cuff injury. However, the number of patients is small and the follow up duration is short. In the future, multicenter randomized controlled study with a larger population can be conducted.

## Conclusion

In summary, operative treatment can effectively lessen severity of pain in the patients, improve shoulder joint function, increase the range of motion of the shoulder joint and enhance the QoL in treating anterosuperior rotator cuff injury. However, repair of Lafosse I subscapularis injury brings no benefit compared to debridement in treating anterosuperior rotator cuff injury.

## Data Availability

The datasets generated and analyzed during the current study are available from the corresponding author on reasonable request.

## Abbreviations

QoL: Quality of life
VAS: Visual analog scale
ASES: American Shoulder and Elbow Surgeons Shoulder Score
EQ-5D: EuroQol- 5 Dimension
